# SOH estimation for lithium-ion batteries using the distribution of relaxation time and feature optimized multilayer perceptron

**DOI:** 10.1016/j.isci.2025.113443

**Published:** 2025-08-26

**Authors:** Fang Wang, Shiqiang Liu, Shiqin Chen, Qi Zhang, Dafang Wang, Xiaole Ma, Xiaoqian Dai

**Affiliations:** 1 China Automotive Technology & Research Center Co. Ltd, Tianjin 300300, China; 2 School of Automotive Engineering, Harbin Institute of Technology, Weihai, Shandong 264209, China

**Keywords:** Applied computing, Energy engineering, Materials science

## Abstract

Electrochemical impedance spectroscopy (EIS) offers abundant dynamic information related to battery degradation. However, the distribution of relaxation time (DRT) methods often produces ambiguous timescale features due to overlapping peaks and the subjective selection of regularization parameters. We propose a DRT method based on RQ elements. This approach significantly reduces parameter sensitivity and enhances the stability of extracted features. The most informative peaks and their combinations are optimized using a feature selection algorithm and are then used as inputs to a multilayer perceptron (MLP) for SOH estimation. The proposed method is validated on two commercial LIB chemistries under different states of charge (SOC) as well as on an open-access dataset. The results demonstrate that the health indicators extracted via the RQ-DRT method and feature combinations effectively reduce redundancy and improve SOH estimation performance. By bridging impedance-based timescale analysis and machine learning, this work provides a practical pathway for SOH monitoring in battery management systems.

## Highlights

• An innovative DRT method based on RQ elements is proposed
• Pronounced relaxation time distribution function is used for feature extraction
• The feature combinations were analyzed based on the MRMR algorithm
• The proposed method is effective under various SOC for multiple batteries

## Graphical abstract

**Figure:**
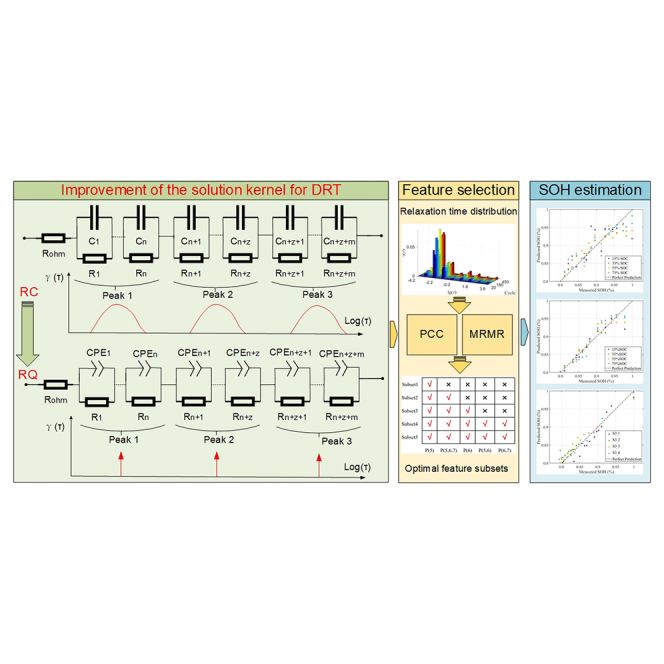

## Introduction

Driven by factors such as carbon neutrality and energy shortages, electric vehicles have rapidly advanced.^1^^,^^2^ Lithium-ion batteries (LIBs), with their high energy density, high power density, and long cycle life, have become the primary power source for electric vehicles.^3^^,^^4^ Extensive research has been conducted on the performance of LIBs; however, concerns regarding their safety and reliability remain unresolved.^5^^,^^6^^,^^7^ LIBs inevitably degrade during use, leading to declines in capacity and power. Understanding the degradation mechanisms and assessing the state of health (SOH) of LIBs are essential for developing new health-aware battery management systems (BMS).^8^^,^^9^ Accurate SOH information enables the reliable estimation of state of charge (SOC), state of power (SOP), and other key metrics, helping to ensure LIBs' reliability while effectively reducing potential hazards.^10^^,^^11^

Current SOH estimation methods are primarily divided into two categories: model-based methods^12^^,^^13^^,^^14^ and data-based methods.^15^^,^^16^^,^^17^ Model-based methods primarily rely on electrochemical models^14^^,^^18^ and equivalent circuit models,^12^^,^^19^ establishing the relationship between model parameters and SOH by identifying parameters under various aging conditions. The advantage of model-based methods lies in the clear physical meaning of model parameters, as they can rely on kinetic information for a more reliable estimation of SOH. However, obtaining model parameters under different aging states requires substantial effort, and parameter accuracy cannot always be guaranteed. Additionally, the SOH estimation performance of model-based methods depends on operating conditions and the suitability of the model, limiting their generalizability.^20^^,^^21^ With the ease of data acquisition and advances in machine learning (ML), data-based methods have attracted considerable attention in recent years. A substantial amount of experimental data and powerful intelligent algorithms facilitate accurate SOH estimation. Meanwhile, some researchers have developed model-data fusion methods by integrating the advantages of models and data-driven approaches.^22^ The accuracy of data-driven methods depends on the extraction and selection of health indicators (HIs).^23^^,^^24^^,^^25^

HIs can be extracted from various types of data, such as current,^26^ voltage,^27^ temperature,^16^^,^^28^ force,^29^ impedance,^30^ and ultrasound.^31^ Voltage-based studies are the most widely used, including HIs extraction from relaxation voltage curves^32^ and charge voltage curves.^33^^,^^34^^,^^35^^,^^36^ Additionally, some signal processing techniques, such as incremental capacity analysis (ICA) and differential voltage analysis (DVA), are frequently used to identify HIs from voltage data.^19^^,^^37^^,^^38^^,^^39^ However, extracting HIs from voltage data typically requires specific current-voltage conditions. For instance, voltage curves used in ICA and DVA analysis need to be obtained at low current rates, with certain restrictions on voltage ranges to capture peaks and valleys. Moreover, in practical applications, both the initial and termination SOCs during battery charging are often not fixed, which limits the applicability of SOH estimation using constant-voltage (CV) charge segments.^32^ After extracting HIs, selecting an appropriate set of HIs is crucial to SOH estimation performance. Including HIs from additional dimensions, such as voltage and temperature, does not always enhance performance. It can also have a negative impact, slowing computation and reducing model interpretability.^15^

In addition to extracting HIs from time-domain information, impedance-based HIs have gained more attention in recent years.^12^^,^^19^^,^^30^^,^^40^^,^^41^ Electrochemical impedance spectroscopy (EIS) contains rich kinetic information, and studies have shown that SOH is highly correlated with these kinetic characteristics. When combined with ML methods such as Gaussian process regression (GPR), neural networks (NN) and XGBoost, superior SOH estimation performance can often be achieved.^23^^,^^30^^,^^41^^,^^42^ At the same time, the development of methods for obtaining in-vehicle EIS has laid the foundation for the application of EIS in real-world vehicles.^43^^,^^44^ The extraction of HIs from EIS can be divided into direct methods and indirect methods. The direct method refers to directly obtaining impedance and phase angle at specific frequencies from the EIS, thereby establishing a link to SOH.^45^^,^^46^ However, the direct method lacks universality, as the type of LIBs, temperature, and aging state can significantly influence the shape of the impedance spectrum. The indirect method involves first parameterizing the EIS through some processing techniques or extracting timescale information before proceeding to HIs extraction. The FOM model, which is commonly used for fitting EIS with equivalent circuit models, estimates SOH by obtaining the variation patterns of model parameters from fitting EIS at different SOH.^12^^,^^47^ The distribution of relaxation time (DRT) is a powerful method that requires no prior knowledge and decouples EIS based on the different time constants of various kinetic processes, allowing for the extraction of relevant HIs.^23^^,^^41^ However, when processing EIS using DRT, it is crucial to ensure the reliability and analyzability of the extracted timescale information. Since the deconvolution of the DRT is an ill-posed problem, regularization techniques are commonly employed, with Tikhonov regularization being the most widely used.^48^ To obtain satisfactory DRT reconstruction results, researchers often need to iteratively fine-tune the regularization parameter.^49^ To mitigate the influence of manual tuning, Li introduced an elastic net regularization approach that automatically determines the optimal parameter.^50^ Additionally, Zhang proposed a variant of Tikhonov regularization that eliminates the need for parameter adjustment,^51^ and Gavrilyuk further analyzed its frequency resolution and robustness against noise.^52^ While these efforts have significantly improved regularization strategies, most studies remain focused on refining the regularization techniques themselves. To the best of our knowledge, no prior work has explored enhancing the DRT inversion by modifying the underlying DRT kernel, which we propose as a novel direction in this study.

Based on the analysis above, this article focuses on a method that can obtain more stable impedance-based timescale information, and achieves superior SOH estimation performance through feature selection methods and a multilayer perceptron (MLP). The main contributions of this article can be summarized as follows: 1) To make the impedance-based timescale information more representative, DRT method based on RQ elements is developed, which makes the peaks of the relaxation time distribution function more conducive to feature extraction; 2) The correlation coefficient method and MRMR algorithm are used to select appropriate HIs and combine feature subsets from different dimensions; 3) Based on the selected HIs, MLP achieves high-performance SOH estimation across three types of LIBs and four different SOCs, while three different regression algorithms are also employed to validate the effectiveness of the feature selection results. This work emphasizes a novel EIS-based feature extraction strategy, which, when integrated with systematic feature selection and SOH estimation, forms a comprehensive framework guided by both physical insight and data-driven techniques.

## Results

### Experiment dataset

The batteries used in this study include two types of commercial batteries: DLG 18650 and Tesla 21700. The cathode material of the DLG 18650 battery is nickel-cobalt-manganese, with a rated capacity of 3.2Ah and a working voltage range from 2.5V to 4.2V. The cathode material of the Tesla 21700 battery is nickel-cobalt-aluminum, with a rated capacity of 4.8Ah and a working voltage range from 2.5V to 4.2V. The detailed parameters of the batteries are shown in Table 1.

**Table 1:** Parameters of the batteries under the test

Parameter	S1	S2	S3(Open-access dataset)
Manufacturer	DLG	Tesla	Eunicell
Nominal capacity	3.2Ah	4.8Ah	45mAh
Operating window	2.5V–4.2V	2.5V–4.2V	3V–4.2V
Chemistry	NCM	NCA	LCO

To obtain aging data for the batteries, accelerated aging tests at high temperatures and reference performance tests at room temperature were conducted. The specific experimental procedure is as follows:

(1) Before the aging process begins, capacity tests, EIS tests, and dynamic condition tests are performed at 25°C as reference performance tests. EIS tests were conducted using the Zennium Pro system (Zahner, Germany), with a frequency range spanning from 0.01 Hz to 1 kHz. A sinusoidal current excitation method was employed, with the root-mean-square amplitude of the perturbation current set to 100 mA. For battery samples S1 and S2, EIS measurements were performed under four different SOCs. Prior to each EIS test, the battery SOC was adjusted to the target value and allowed to rest for 1 h to ensure electrochemical equilibrium. This step is crucial to ensure that the EIS measurements satisfy the fundamental prerequisites of causality, linearity, and stability.
(2) The temperature chamber is adjusted to 40°C. After the battery reaches thermal equilibrium, it is first charged to 4.2V using a constant current of 3C, followed by constant voltage charging until the current drops to 0.05C. After standing for 10 min, the battery is discharged to 2.5V using a constant current of 3C, followed by another 10-min rest. The constant current charging, constant voltage charging, rest, constant current discharging, and rest phases together constitute one aging cycle. A total of 20 aging cycles constitutes one round of aging tests.
(3) After completing one round of aging tests, the temperature of the chamber is returned to 25°C, and a new round of reference performance tests is conducted on the battery. The SOH of the battery is determined based on the remaining capacity. If the SOH falls below 80%, the aging test is terminated; if the SOH remains above 80%, the next round of aging tests is conducted until the SOH falls below 80%.

In addition, our proposed method was validated using the open-access EIS dataset provided by the University of Cambridge.^53^ The battery cells used in this dataset are summarized in Table 1. This dataset records the EIS of coin cells at various states throughout each cycle. Among these states, the one labeled as “StateV” corresponds to the fully charged condition. It has been widely adopted in the literature for estimating SOH due to its superior stability and reliability in EIS measurements compared to other states.^54^^,^^55^^,^^56^ In our study, we selected the EIS data of four samples, specifically 25C01, 25C02, 25C03, and 25C05, under the StateV condition. For clarity in presentation and analysis, these samples were renamed as S3_1, S3_2, S3_3, and S3_4, respectively.

In this study, SOH of the battery is defined as the percentage of the remaining capacity of the battery relative to its initial capacity, as shown below:

(Equation 1) $$SOH=\frac{C_{cyc}}{C_{ori}}\times 100\%$$

**Figure 1:**
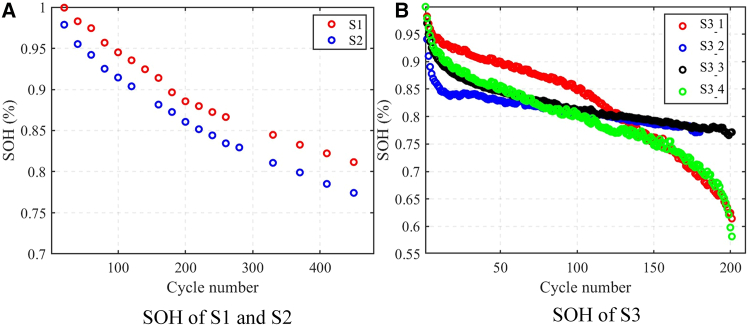
The measured SOH (A) SOH of S1 and S2 (B) SOH of S3.

The SOH of S1 and S2 as a function of aging cycles is shown in the Figure 1A. It can be observed that with the increase in the number of cycles, the SOH of S2 decreases at a faster rate compared to S1, which may be due to differences in the internal material systems of the batteries. During the battery aging process, we conducted EIS tests on the batteries at four different SOC levels (15%, 35%, 55%, and 75%).

**Figure 2:**
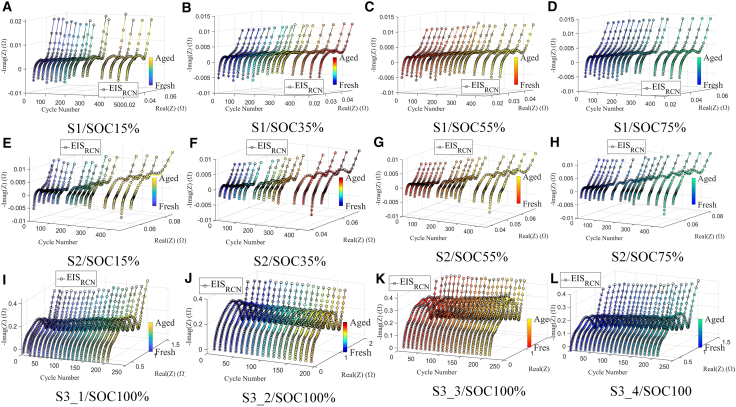
Comparison between the reconstructed EIS and measured EIS (A) S1/SOC15% (B) S1/SOC35% (C) S1/SOC55% (D) S1/SOC75% (E) S2/SOC15% (F) S2/SOC35% (G) S2/SOC55% (H) S2/SOC75% (I) S3_1/SOC100% (J) S3_2/SOC100% (K) S3_3/SOC100% (L) S3_4/SOC100.

The test results of S1 and S2 are shown in Figures 2A–2H. It can be observed that with the increase in the number of cycles, both S1 and S2 show an increase in the size of the semicircles in the middle and high-frequency regions, with the change in S2 being more pronounced compared to S1. The semicircles in the middle and high-frequency regions are related to the charge transfer effect inside the battery, and their impedance shows more significant changes during aging, which is consistent with previous research findings. However, despite this, it remains challenging to quantify the relationship between the information in the electrochemical impedance spectrum and SOH. Therefore, model fitting or decoupling algorithms are needed to further extract more effective information.

The evolution of EIS with battery aging for samples S3_1 to S3_4 is presented in Figures 2I–2L. While the extent of variation differs across the four cells, all exhibit a noticeable increase in charge transfer resistance in the mid-frequency region as aging progresses. This trend indicates a common electrochemical degradation pattern. However, directly using raw EIS data for SOH estimation remains challenging due to its high dimensionality and the lack of clearly distinguishable features. Therefore, it is essential to investigate effective EIS parameterization approaches that can extract informative features to improve the accuracy and interpretability of SOH estimation.

### Distribution of relaxation time analysis results

**Figure 3:**
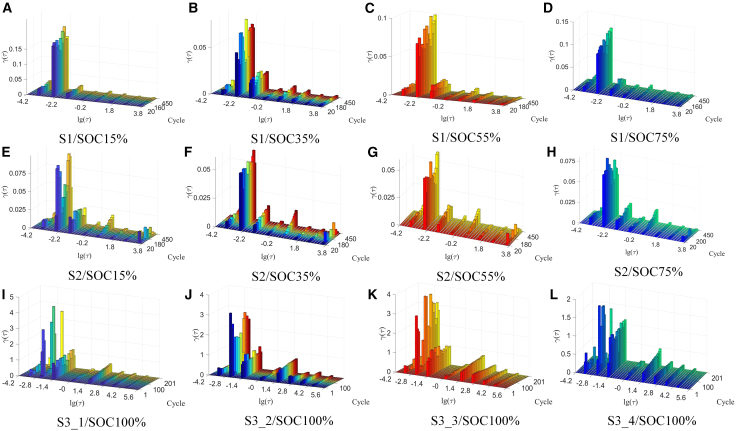
The relaxation time distribution function (A) S1/SOC15% (B) S1/SOC35% (C) S1/SOC55% (D) S1/SOC75% (E) S2/SOC15% (F) S2/SOC35% (G) S2/SOC55% (H) S2/SOC75% (I) S3_1/SOC100% (J) S3_2/SOC100% (K) S3_3/SOC100% (L) S3_4/SOC100%.

To make the peaks of the relaxation time distribution function more convenient for analysis and to extract relevant information for SOH estimation, this study proposes an RQ element-based DRT analysis method in Section 2. DRT solutions were applied to the EIS of S1-S3 at different SOCs and aging states to obtain their relaxation time distribution functions. Figure 3 presents the results of the relaxation time distribution functions at different SOCs and aging states. It can be seen that all types of batteries exhibit eight distinct peaks. Compared to the relaxation time distribution functions obtained from the DRT analysis based on RC elements, the functions derived using the RQ element-based DRT method are more concentrated around fixed time constants, making it easier to analyze the peaks as a function of aging state. We classify the peaks based on the logarithm of the time constants in increasing order, and label them as P1-8. According to the distribution patterns of the relaxation time distribution functions for different electrochemical processes,^40^ P1-4 correspond to the solid-liquid phase diffusion within the battery; P5-7 correspond to the impedance of charge transfer processes and the impedance of the SEI film; and P8 corresponds to the contact impedance between the current collector and the active material. The visualization analysis of the eight peaks reveals distinct aging-related behaviors. Peaks P1 to P4 exhibit no consistent trend with aging across the three different battery types. In contrast, P5 shows a clear and continuous increase in all samples, which aligns with its electrochemical interpretation as representing charge transfer resistance. The increase in charge transfer resistance with aging is well documented in the literature and reflects the progressive degradation of electrochemical kinetics. Peak P6 displays divergent trends depending on the sample. For S1 and S2, P6 does not exhibit a clear linear pattern. However, in the S3 samples, P6 consistently decreases with aging. Since P6 corresponds to the resistance of the solid electrolyte interphase (SEI) layer, this decrease appears counterintuitive. Normally, the SEI layer becomes thicker during aging, which would typically lead to an increase in SEI resistance. The observed reduction in SEI resistance for S3 may be attributed to variations in SEI composition resulting from different aging temperatures and current rates. These conditions could produce new SEI species with higher ionic conductivity, thereby reducing the overall resistance.^57^ Peaks P7 and P8 do not exhibit any significant linear trend during the aging process, suggesting limited relevance to aging-related impedance evolution.

After obtaining the relaxation time distribution function, we can verify its accuracy by performing the inverse DRT transformation. The DRT inverse transformation involves using the obtained relaxation time distribution function to reconstruct the EIS, also known as the EIS_RCN_. The accuracy of the relaxation time distribution function solution can be verified by comparing the reconstructed EIS with the measured EIS in terms of their similarity. As shown in Figure 2, for S1-S3 at different SOCs and various aging states, the RQ-element-based DRT method successfully fits the measured EIS, indicating that the solution for the relaxation time distribution function is reliable.

### Feature extraction results

By solving the relaxation time distribution function of EIS using the RQ element-based DRT, we can obtain eight peaks with similar time constants. To estimate SOH using these eight peaks, it is necessary to further analyze the linear relationships between the peaks and SOH. Additionally, considering the time constant ranges of different electrochemical processes, we also consider combinations of peaks representing the same electrochemical process and their linear relationships with SOH. The selection of parameters highly linearly correlated with SOH, based on Pearson correlation coefficients, is shown in Table 2.

**Table 2:** The correlation coefficients between different peaks or peak combinations and SOH

Cell	SOC	1	2	3	4	5	6	7	8	(1,2,3,4)	(5,6)	(6,7)	(5, 6, 7)
S1	15%	−0.26	−0.27	0.14	−0.40	**−0.79**	0.04	0.17	0.05	−0.26	**−0.81**	0.11	**−0.71**
	35%	−0.47	−0.48	−0.15	−0.47	**−0.86**	−0.53	0.25	−0.45	−0.58	**−0.92**	−0.39	**−0.89**
	55%	−0.66	−0.65	0.13	−0.29	**−0.77**	−0.44	−0.36	0.17	−0.74	**−0.89**	−0.55	**−0.92**
	75%	−0.64	−0.60	−0.15	0.46	**−0.93**	−0.23	−0.27	−0.13	−0.72	**−0.94**	−0.39	**−0.94**
S2	15%	−0.10	−0.20	0.10	−0.84	**−0.95**	0.07	0.51	0.17	−0.20	**−0.92**	0.27	**−0.91**
	35%	−0.41	−0.33	−0.44	−0.66	**−0.96**	−0.34	0.03	0.08	−0.55	**−0.93**	−0.26	**−0.92**
	55%	−0.30	−0.22	−0.35	−0.73	**−0.95**	0.12	0.30	0.03	−0.38	**−0.96**	0.33	**−0.94**
	75%	0.36	0.31	−0.52	0.25	**−0.98**	−0.04	0.36	0.44	0.22	**−0.96**	0.14	**−0.95**
S3_1	100%	−0.05	0.04	−0.20	0.11	**−0.98**	**0.95**	0.49	0.22	0.22	**−0.98**	**0.95**	**−0.97**
S3_2	100%	0.33	0.23	−0.29	−0.09	**−0.87**	**0.83**	0.01	0.71	0.14	0.39	**0.80**	0.26
S3_3	100%	−0.03	−0.16	−0.07	−0.36	**−0.90**	**0.80**	0.67	0.79	−0.25	**−0.91**	**0.83**	**−0.91**
S3_4	100%	0.47	0.11	−0.61	0.04	**−0.98**	**0.86**	0.72	−0.29	−0.17	**−0.97**	**0.84**	**−0.96**

Bold indicate strong relevance.

As shown in Table 2, peak P5 demonstrates a strong correlation with SOH across all three battery types, which is consistent with the trends observed directly from the DRT function. In addition, peak P6 shows a strong positive correlation with SOH for the S3 batteries, a relationship that is not observed in S1 and S2. The combined features P(5,6) and P(5,6,7), which represent the contributions from both charge transfer resistance and SEI layer resistance, generally exhibit strong correlations with SOH. The only exception is S3_2, where this correlation is not significant. This is attributed to the relatively small increase in charge transfer resistance and the pronounced decrease in SEI resistance in S3_2, as observed in its DRT profile. Furthermore, the combination of peaks P(6,7), corresponding to two components of SEI layer resistance, consistently shows a strong positive correlation with SOH across all four S3 cells. Based on the Pearson correlation analysis of individual peaks and peak combinations, P5, P(5,6), and P(5,6,7) are selected as HIs for S1 and S2. For the S3 batteries, P5, P6, P(5,6), P(6,7), and P(5,6,7) are selected as HIs, considering their strong correlation with the battery’s SOH.

**Figure 4:**
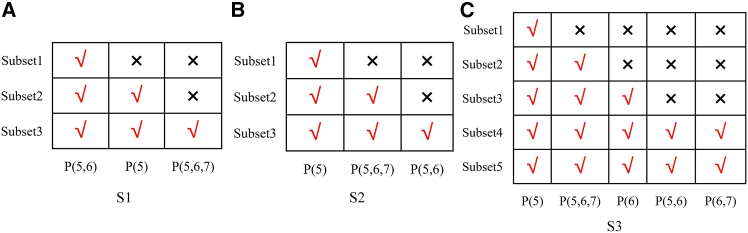
The optimal feature subsets (A) S1, (B) S2, and (C) S3.

Since the selected HIs consist of both individual peaks and their combinations, it is necessary to avoid introducing redundant information during the SOH estimation process. To address this, the MRMR algorithm is employed to rank the importance of the selected features. The MRMR algorithm evaluates feature importance by simultaneously maximizing the relevance between features and the target variable while minimizing redundancy among features. It also enables the selection of optimal feature subsets based on a predefined number of features, which effectively reduces dimensionality and model complexity. The results of feature selection using the MRMR algorithm for S1, S2, and S3 are shown in Figure 4. As illustrated in the figure, the top three features selected for S1 are P(5,6), P5, and P(5,6,7), in descending order of importance. For S2, the three most important features are P5, P(5,6,7), and P(5,6). In the case of S3, the five selected features, ranked by importance, are P5, P(5,6,7), P6, P(5,6), and P(6,7). A more detailed discussion of the feature ranking results based on MRMR is provided in different feature combinations of the discussion.

### State of health estimation results

**Figure 5:**
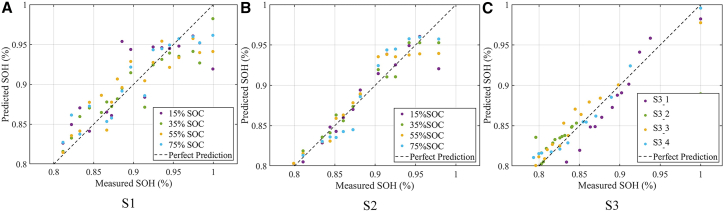
The SOH estimation results based on MLP (A) S1, (B) S2, and (C) S3.

The results of SOH estimation using HIs extracted via the RQ-based DRT method are illustrated in Figure 5. The horizontal axis represents the measured SOH, while the vertical axis denotes the predicted SOH. The dashed line indicates perfect prediction, where the estimated value exactly matches the measured one. As shown in Figures 5A and 5B, the proposed method achieves high prediction accuracy for both S1 and S2 across a wide range of SOC levels, demonstrating the robustness and generalizability of the RQ-DRT-based HIs. For S1, the estimation accuracy is relatively lower during the early aging stages. The prediction points are more scattered around the perfect prediction line, particularly at an SOC of 15% (represented by purple dots), where the deviation is most significant. This indicates that the SOH prediction error is larger at low SOC levels. In contrast, S2 achieves higher estimation accuracy throughout the aging process, with prediction points more closely aligned with the perfect prediction line. This improvement is primarily attributed to the stronger correlation between the extracted HIs and SOH in S2. All selected indicators exhibit Pearson correlation coefficients greater than 0.9 in absolute value, which enhances the reliability of the SOH estimation. Figure 5C further confirms the adaptability of the proposed method to different battery samples. Although larger estimation errors are observed at the beginning of the aging process when SOH is 100%—mainly due to a sharp initial capacity drop in the S3 cells—the prediction error decreases as aging progresses. Similar to S1 and S2, the largest estimation error in S3 also occurs at an SOC of 15%, suggesting that further improvement is needed in model performance under low SOC conditions.

**Figure 6:**
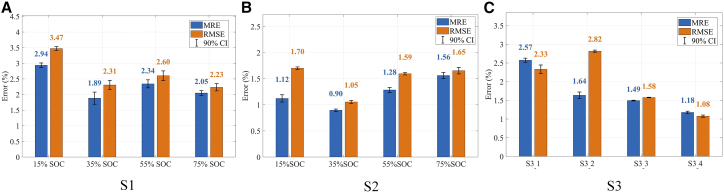
SOH estimation errors (A) S1, (B) S2, and (C) S3.

As shown in Figure 6, the proposed method achieves consistently low SOH estimation errors under all tested conditions, including varying SOC levels (S1 and S2) and different battery cells (S3). In 10 repeated training runs, the MRE of all samples remained below 3%, and the RMSE remained below 3.5%. The lengths of the 90% confidence intervals (CIs) for both MRE and RMSE were within 1%, indicating that the model exhibits strong stability and reliability. Taking RMSE as the evaluation metric: For dataset S1, the highest estimation error occurs at 15% SOC with an RMSE of 3.47%, while the lowest error is observed at 75% SOC with an RMSE of 2.23%; For dataset S2, the largest error also appears at 15% SOC (1.70%), and the most accurate results are achieved at 35% SOC, with an RMSE of only 0.90%; Regarding the four individual cells in dataset S3, cell S3_2 yields the largest estimation error (2.82%), whereas cell S3_4 achieves the best accuracy with an RMSE of 1.08%.The average RMSE on the open dataset is 1.72%. Compared with existing studies using the same open-access dataset, the accuracy achieved in this work is highly competitive.^54^^,^^56^ More detailed error information is summarized in Table 3, allowing for an intuitive comparison of the model's performance in different scenarios from the table. Overall, the proposed method demonstrates competitive SOH estimation performance with strong robustness across different SOC conditions and battery cells.

**Table 3:** SOH estimation performance

Samples	Feature	MPE (%)	MPE_90%CI (%)	RMSE (%)	RMSE_90%CI(%)
S1	15% SOC	2.94	0.07	3.47	0.06
	35% SOC	1.89	0.19	2.31	0.15
	55% SOC	2.34	0.13	2.60	0.15
	75% SOC	2.05	0.08	2.23	0.12
	Average	2.30	0.54	2.65	0.67
S2	15% SOC	1.12	0.07	1.70	0.02
	35% SOC	0.90	0.02	1.05	0.03
	55% SOC	1.28	0.05	1.59	0.02
	75% SOC	1.56	0.06	1.65	0.06
	Average	1.22	0.33	1.50	0.35
S3	S3_1	2.57	0.05	2.33	0.11
	S3_2	1.64	0.09	2.82	0.03
	S3_3	1.49	0.01	1.58	0.01
	S3_4	1.18	0.03	1.08	0.03
	Average	1.72	0.71	1.95	0.91

## Discussion

### Different feature combinations

**Figure 7:**
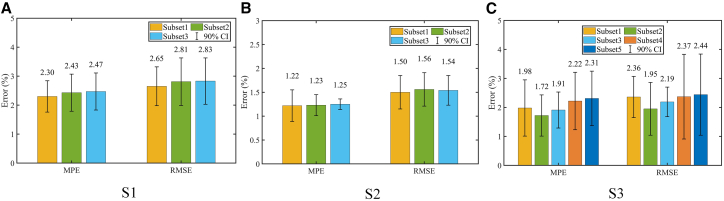
The impact of feature subset (A) S1, (B) S2, and (C) S3.

Properly controlling the number of input features not only reduces model complexity but also enhances the accuracy of SOH estimation. In this study, the MRMR algorithm was employed to select three optimal feature subsets for datasets S1 and S2, and five subsets for dataset S3. To evaluate how different combinations of features affect the prediction performance, we trained models using each subset. The results are illustrated in Figure 7, which presents the MPE and RMSE for each case. As shown in Figures 7A and 7B, a slight increase in SOH estimation error is observed as the number of selected features increases for S1 and S2. This trend indicates that including more features may introduce redundancy, thereby impairing the estimation accuracy. In contrast, for S3 in Figure 7C, Subset2 achieves the lowest estimation error, suggesting that the selected features (P5 and P(5,6,7)) exhibit high relevance and minimal redundancy. Overall, the comparative results across different feature subsets support the conclusion that the MRMR algorithm is effective in selecting representative features under redundancy conditions, leading to improved performance in SOH regression tasks.

### Different regression algorithms

To comprehensively validate the performance of MLP in SOH estimation tasks, we also employed three commonly used regression models, namely linear regression (LR), Support Vector Machine (SVM), and Gaussian Process Regression (GPR), for comparative analysis. All models used the optimal feature subsets selected through the MRMR algorithm as input and were trained and evaluated using 4-fold cross-validation. In order to objectively compare the prediction performance among different algorithms, MPE and RMSE were adopted as the primary evaluation metrics. Additionally, the 90% confidence interval length was introduced to assess the stability of each model. The SVM model was implemented with a radial basis function (RBF) kernel, where the penalty parameter C was set to 10 and the epsilon-insensitive loss function parameter ε was set to 0.01. The LR model was implemented using standard ordinary least squares without regularization. The GPR model employed a composite kernel consisting of a constant kernel multiplied by a Matérn kernel (ν = 1.5, length scale = 1.0), plus a white noise kernel (noise level = 1e-2). The model also used α = 1 × 10−3, enabled output normalization, and performed 10 optimizer restarts to improve convergence. All hyperparameters were selected through grid search to ensure optimal performance.

**Figure 8:**
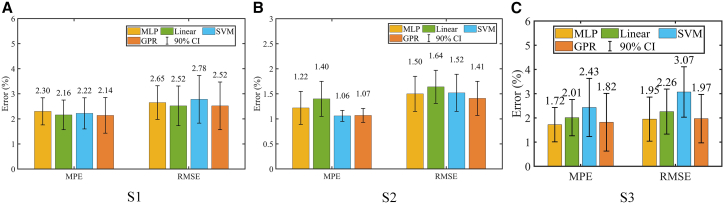
The impact of regression algorithms (A) S1, (B) S2, and (C) S3.

The estimation errors on datasets S1, S2, and S3 are illustrated in Figure 8. As shown in the figure, on datasets S1 and S2, the MLP model does not exhibit the highest accuracy. For instance, on S2, SVM achieved the lowest MAE of 1.06%, while GPR achieved the lowest RMSE of 1.41%. Nevertheless, MLP demonstrated better stability on both datasets, with relatively narrower confidence intervals. In particular, the RMSE_ci90 of MLP on S1 was only 0.67%, which is notably smaller than the 0.95% of SVM. On dataset S3, MLP achieved both the lowest MAE of 1.72% and the lowest RMSE of 1.95%, while also maintaining a relatively small confidence interval. This result indicates that MLP has strong generalization ability across different battery cells.

To further understand these results, we analyze the error patterns based on the characteristics of the models and the SOH estimation task. The superior performance of SVM on dataset S2 can be attributed to its strong local fitting capability, particularly when the input–output relationship is smooth and well-structured. However, SVM may suffer from overfitting or instability when encountering higher noise or nonuniform feature relevance, as observed in its wider confidence intervals on dataset S1. GPR, on the other hand, benefits from its probabilistic nature and inherent ability to quantify uncertainty. It performs well on datasets with moderate data size and consistent noise levels, which explains its low RMSE on S2. However, due to its kernel-based formulation and computational complexity, GPR may struggle with generalization in more heterogeneous datasets such as S3, leading to less stable performance across folds. MLP, as a flexible and highly parameterized model, may not always achieve the absolute lowest error in every case. However, its ability to learn complex nonlinear relationships across a broader feature space contributes to its robust generalization, particularly when trained with well-selected features. Its lower variation and narrower confidence intervals across all three datasets, especially on the more inconsistent dataset S3, highlight its adaptability and robustness under varying data distributions and battery chemistries. These observations suggest that while simpler models may occasionally outperform MLP in specific settings, MLP offers a more balanced trade-off between accuracy and stability, which is crucial for practical SOH estimation across diverse operating conditions.

### Conclusion

This study presents an RQ-based DRT approach for extracting reliable HIs from EIS data to estimate the SOH of lithium-ion batteries. By replacing the traditional RC element with the more physically representative RQ element, the proposed method yields smoother and more analyzable relaxation time distributions, reducing pseudo-peaks and improving feature stability. The MRMR algorithm is used to select the most relevant features, which are then fed into an MLP model for regression. Experimental results on two types of commercial LIBs (NCM and NCA) and an open dataset demonstrate that the proposed method achieves high estimation accuracy across different SOC levels (15%–75%). Specifically, the mean percentage error remains below 2.3%, and the RMSE does not exceed 2.6%, with 90% confidence interval lengths consistently under 1%. Compared with linear regression, SVM, and Gaussian process regression, the MLP model offers better overall performance, particularly for battery S3, where it achieves the lowest RMSE and MAE with minimal variation across runs. In summary, the integration of RQ-based DRT, MRMR feature selection, and MLP modeling provides a robust and interpretable framework for SOH estimation. The method shows strong generalizability and stability under varying battery chemistries and operating conditions, indicating its potential for practical application in battery management systems.

Looking forward, the proposed method shows strong potential for deployment in battery pack applications. With recent advances in online impedance measurement techniques that enable cell-level EIS acquisition within modules,^58^^,^^59^ our approach could be seamlessly integrated into next-generation BMSs for real-time SOH monitoring. Moreover, as demonstrated on datasets with evident cell inconsistency, our model maintains robust SOH estimation accuracy, indicating its suitability for practical pack-level applications where heterogeneity among cells is common.

### Limitations of the study

Despite the promising results and demonstrated the generalizability of the proposed method, several limitations remain that warrant further investigation: 1) Limited diversity of battery chemistries: Although the method has been validated on two types of commercial lithium-ion batteries and an open-access dataset, it lacks evaluation on lithium iron phosphate (LFP) batteries, which are widely used in energy storage and electric vehicles. Future work should incorporate a broader range of battery chemistries and manufacturers to ensure wider applicability. 2) Ordinary aging conditions: The aging scenarios considered in this study primarily reflect standard usage conditions. However, real-world applications often involve harsh or extreme environments, as well as unexpected damage or stress-induced degradation. To enhance the robustness and practical relevance of the model, future studies should include batteries aged under extreme temperatures, high-rate cycling, overcharging, or mechanical abuse. 3) Pack-level adaptation and inconsistency analysis: While the method shows potential for application at the battery pack level, further algorithmic development is needed to address the challenges posed by cell-to-cell inconsistencies within modules and packs. Developing scalable SOH estimation frameworks that can accommodate individual cell variations and spatial heterogeneity will be crucial for real-world deployment in battery management systems.

## Resource availability

### Lead contact

Further information and requests for resources and reagents should be directed to and will be fulfilled by the Lead Contact, Shiqin Chen (23b908096@stu.hit.edu.cn).

### Materials availability

This study did not generate new materials.

### Data and code availability

• The battery dataset used in this study comes from Zhang et al. and is available at https://doi.org/10.5281/zenodo.3633835.
• All original code has also been deposited at https://github.com/ccqq27/Enhanced-SOH-Estimation-for-Lithium-Ion-Batteries.git and is publicly available, including the RQ-DRT algorithm for extracting body features and the algorithm for SOH estimation.
• Any additional information required to reanalyze the data reported in this article is available from the lead contact upon request.

## Acknowledgments

This work was financially supported by 10.13039/501100012166National Key R&D Program of China (Grant No. 2023YFB2503803) and the Natural Science Youth Foundation of Shandong Province (ZR2024QB165).

## Author contributions

Fang wang: supervision, funding acquisition, resources, and project administration. Shiqiang Liu: validation, data curation, and writing – review. Shiqin Chen: conceptualization, software, validation, visualization, writing – original draft, and writing – review and editing. Qi Zhang: data curation, validation, and visualization. Dafang Wang: data curation, resources, and project administration. Xiaole Ma: data curation and validation. Xiaoqian Dai: validation and visualization.

## Declaration of interests

The authors declare no competing interests.

## STAR★Methods

### Key resources table

**Table:** 

REAGENT or RESOURCE	SOURCE	IDENTIFIER
**Deposited data**		

45mAh Eunicell LR2032	Yunwei Zhang	Zenodo:3633835 (https://doi.org/10.5281/zenodo.3633835)

**Software and algorithms**		

MATLAB 2020b	MATLAB	https://ww2.mathworks.cn/products/matlab.html
Python	Python Software Foundation	https://www.python.org/
RQ-DRT	This paper	Github:ccqq27 (https://github.com/ccqq27/Enhanced-SOH-Estimation-for-Lithium-Ion-Batteries.git)
SOH estimation	This paper	Github:ccqq27 (https://github.com/ccqq27/Enhanced-SOH-Estimation-for-Lithium-Ion-Batteries.git)

### Method details

#### RQ elements-based DRT

The DRT is a commonly used method for decoupling EIS, enabling the transformation of frequency-domain impedance into the time domain to obtain relaxation time distribution information for different electrochemical processes. The DRT method can be divided into two main components: the solution kernel and the solution method. The solution kernel refers to the combination of circuit elements used to approximate the EIS curve, while the solution method denotes the approach used to solve the parameters within the solution kernel. For EIS curves at different frequency ranges, various solution kernels are available. The most common solution kernels include ohmic resistance, inductive elements, and multiple RC elements,^42^ with the impedance expression given as follows:

(Equation 2) $$Z_{sim}=R_{0}+j\omega L_{0}+\sum_{i}^{N_{DRT}}\frac{\gamma \left(\tau_{i}\right)}{1\;+\;j\omega \tau_{i}},\tau_{i}=R_{i}C_{i}$$

where, $R_{0}$ is ohmic resistance, $L_{0}$ is inductance in test leads and the battery, $N_{DRT}$ is the number of RC elements used for DRT analysis, $\tau_{i}$ is the time constants of $N_{DRT}$ RC elements. Typically, each RC element has a specific and continuous relaxation time. RC elements with similar relaxation times are grouped together to represent a single electrochemical reaction. $\gamma \left(\tau_{i}\right)$ is relaxation time distribution function。

By separating the real and imaginary components of the impedance in Equation 2, we can transform the solution for the distribution function, ohmic resistance, and inductance into the form Ax = b, as shown in Equation 3:

(Equation 3) $$\left[\begin{array}{ccccc} \frac{1}{1\;+\;\omega_{1}^{2}\tau_{1}^{2}} & \cdots & \frac{1}{1\;+\;\omega_{1}^{2}\tau_{N_{DRT}}^{2}} & 1 & 0\\ \vdots & \ddots & \vdots & \vdots & \vdots \\ \frac{1}{1\;+\;\omega_{N_{EIS}}^{2}\tau_{1}^{2}} & \cdots & \frac{1}{1\;+\;\omega_{N_{EIS}}^{2}\tau_{N_{DRT}}^{2}} & 1 & 0\\ \frac{-\omega_{1}\tau_{1}}{1\;+\;\omega_{1}^{2}\tau_{1}^{2}} & \cdots & \frac{-\omega_{1}\tau_{N_{DRT}}}{1\;+\;\omega_{1}^{2}\tau_{N_{DRT}}^{2}} & 0 & \omega_{1}\\ \vdots & \ddots & \vdots & \vdots & \vdots \\ \frac{-\omega_{N_{EIS}}\tau_{1}}{1\;+\;\omega_{N_{EIS}}^{2}\tau_{1}^{2}} & \cdots & \frac{-\omega_{N_{EIS}}\tau_{N_{DRT}}}{1\;+\;\omega_{N_{EIS}}^{2}\tau_{N_{DRT}}^{2}} & 0 & \omega_{N_{EIS}} \end{array}\right]\left[\begin{array}{c} \gamma \left(\tau_{1}\right)\\ \vdots \\ \gamma \left(\tau_{N_{DRT}}\right)\\ R_{0}\\ L_{0} \end{array}\right]=\left[\begin{array}{c} Z_{MEA}^{\text{Re}}\left(\omega_{1}\right)\\ \vdots \\ Z_{MEA}^{\text{Re}}\left(\omega_{N_{EIS}}\right)\\ Z_{MEA}^{\text{Im}}\left(\omega_{1}\right)\\ \vdots \\ Z_{MEA}^{\text{Im}}\left(\omega_{N_{EIS}}\right) \end{array}\right]$$

where, $N_{EIS}$ is the number of measurement points in EIS, $\omega_{i},i=1,2,\cdots N_{EIS}$ is the angular frequency of each measurement point.

The solution for x is actually an ill-posed problem. The nonlinear least squares optimizer used in this study is a commonly employed solver for DRT analysis. Additionally, to make the obtained distribution function smoother and enhance its analyticity, regularization is introduced into the final optimization objective, as shown in Equation 4:

(Equation 4) $$\min\left\{{\left\|Ax-b\right\|}_{2}+\lambda_{T}{\left\|\gamma \left(\tau \right)\right\|}_{2}\right\}$$

where, $\lambda_{T}$ is regularization coefficient, $\lambda {\left\|\gamma \left(\tau \right)\right\|}_{2}$ is penalty term.

The selection of $N_{DRT}$ and $\lambda_{T}$ significantly affects the solution of the relaxation function, as shown in Figure S1. As $N_{DRT}$ increases, the peaks in the distribution function become more distinct, and the spacing between peaks becomes more pronounced, making it easier to analyze each peak individually. However, an increase in $N_{DRT}$ also leads to an increase in computation time, and simply increasing $N_{DRT}$ is not an optimal choice. Based on experience, when $N_{DRT}$ is more than 1.5 times $N_{EIS}$, the error between the DRT fit of $Z_{DRT}$ and $Z_{MEA}$ can be controlled within an acceptable range. Nevertheless, the selection of $N_{DRT}$ must also consider the trade-off between the analyticity of the distribution function and the solution speed. Furthermore, as seen in Figure S1B, as the regularization coefficient $\lambda_{T}$ decreases, the peak features of the distribution function become more distinct, but the points become increasingly less smooth, which may lead to analytical bias. In practical applications, the selection of $N_{DRT}$ and $\lambda_{T}$ is often influenced by subjective factors, which can distort the physical meaning contained in the distribution function.

To obtain a stable and physically interpretable relaxation times distribution function, researchers have long focused on exploring more effective numerical strategies to address the inherent ill-posed of the inverse problem. Conventional approaches for solving the DRT are often influenced significantly by the choice of kernel functions and the regularization coefficients. These factors can introduce artificial peaks in the solution, thereby compromising the reliability of the physical interpretation. To tackle this challenge, this work aims to develop a new type of kernel function that improves the accuracy and consistency of the DRT estimation, ultimately enabling more reliable characterization of underlying electrochemical processes. It is worth noting that the RC element is typically regarded as an idealized lumped-parameter model, which can only offer a simplified approximation of the real system. In contrast, the RQ element provides a more realistic representation of electrochemical systems with distributed relaxation behavior.^60^ The impedance expression of the RQ element is given in Equation 5.

(Equation 5) $$Z_{RQ}\left(\omega \right)=\frac{R}{1\;+\;{\left(j\omega \tau \right)}^{n}},\tau =RQ$$

The impedance characteristics of RC and RQ elements in the Nyquist plot, as well as their corresponding relaxation times distribution function, are illustrated in Figure S2. As shown in the figure, even when the RC and RQ elements exhibit identical impedance spectra, their relaxation times distribution function representations differ markedly. Specifically, the RC element yields two Dirac delta functions in the relaxation times distribution function, whereas the RQ element results in two smooth, continuous peaks. As further illustrated in Figure S1, using RC elements as the kernel for DRT estimation essentially leads to relaxation times distribution function composed of discrete Dirac pulses. This formulation is highly sensitive to the choice of internal kernel function and the regularization coefficient, both of which can significantly alter the amplitude of the Dirac pulses at different frequencies. Such instability often manifests as artificial peaks, which compromise the physical interpretability and reliability of the relaxation times distribution function. In contrast, since the relaxation times distribution function of the RQ element naturally exhibits peak-like features, employing RQ elements as the kernel in DRT estimation promotes a more continuous and physically meaningful relaxation spectrum. This approach not only mitigates the emergence of spurious peaks but also facilitates more accurate extraction of key features, thereby enhancing the interpretability and applicability of the DRT in electrochemical analysis.

To use RQ elements for DRT, the impedance expression for the RQ element must first be integrated into Equation 3. The impedance expression for the RQ element can be transform from Equations 5 and 6:

(Equation 6) $$Z=\frac{R\;+\;R^{2}Q\omega^{n}\;\cos\left(\frac{n\pi }{2}\right)-jR^{2}Q\omega^{n}\;\sin\left(\frac{n\pi }{2}\right)}{1^{2}\;+\;2RQ\omega^{n}\;\cos\left(\frac{n\pi }{2}\right)\;+\;{\left(RQ\omega^{n}\right)}^{2}}$$

The real and imaginary components of the impedance can be written as:

(Equation 7) $$Z_{real}=\frac{R\;+\;R^{2}Q\omega^{n}\;\cos\left(\frac{n\pi }{2}\right)}{1\;+\;2RQ\omega^{n}\;\cos\left(\frac{n\pi }{2}\right)\;+\;{\left(RQ\omega^{n}\right)}^{2}}$$

(Equation 8) $$Z_{imag}=\frac{-jR^{2}Q\omega^{n}\;\sin\left(\frac{n\pi }{2}\right)}{1\;+\;2RQ\omega^{n}\;\cos\left(\frac{n\pi }{2}\right)\;+\;{\left(RQ\omega^{n}\right)}^{2}}$$

To simplify the expression, substitute $RQ=\tau^{n}$ into the equation:

(Equation 9) $$Z_{real}=\frac{1\;+\;{\left(\tau \omega \right)}^{n}\;\cos\left(\frac{n\pi }{2}\right)}{1\;+\;2{\left(\tau \omega \right)}^{n}\;\cos\left(\frac{n\pi }{2}\right)\;+\;{\left(\tau \omega \right)}^{2n}}R$$

(Equation 10) $$Z_{imag}=\frac{-j{\left(\tau \omega \right)}^{n}\;\sin\left(\frac{n\pi }{2}\right)}{1\;+\;2{\left(\tau \omega \right)}^{n}\;\cos\left(\frac{n\pi }{2}\right)\;+\;{\left(\tau \omega \right)}^{2n}}R$$

By integrating Equations 9 and 10 into Equation 3 to replace the RC element, the matrix A can be modified as:

(Equation 11) $$\left[\begin{array}{ccccc} \frac{1\;+\;{\left(\tau_{1}\omega_{1}\right)}^{n}\;\cos\left(\frac{n\pi }{2}\right)}{1\;+\;2{\left(\tau_{1}\omega_{1}\right)}^{n}\;\cos\left(\frac{n\pi }{2}\right)\;+\;{\left(\tau_{1}\omega_{1}\right)}^{2n}} & \cdots & \frac{1\;+\;{\left(\tau_{N_{RQ}}\omega_{1}\right)}^{n}\;\cos\left(\frac{n\pi }{2}\right)}{1\;+\;2{\left(\tau_{N_{RQ}}\omega_{1}\right)}^{n}\;\cos\left(\frac{n\pi }{2}\right)\;+\;{\left(\tau_{N_{RQ}}\omega_{1}\right)}^{2n}} & 1 & 0\\ \vdots & \ddots & \vdots & \vdots & \vdots \\ \frac{1\;+\;{\left(\tau_{1}\omega_{N_{EIS}}\right)}^{n}\;\cos\left(\frac{n\pi }{2}\right)}{1\;+\;2{\left(\tau_{1}\omega_{N_{EIS}}\right)}^{n}\;\cos\left(\frac{n\pi }{2}\right)\;+\;{\left(\tau_{1}\omega_{N_{EIS}}\right)}^{2n}} & \cdots & \frac{1\;+\;{\left(\tau_{N_{RQ}}\omega_{N_{EIS}}\right)}^{n}\;\cos\left(\frac{n\pi }{2}\right)}{1\;+\;2{\left(\tau_{N_{RQ}}\omega_{N_{EIS}}\right)}^{n}\;\cos\left(\frac{n\pi }{2}\right)\;+\;{\left(\tau_{N_{RQ}}\omega_{N_{EIS}}\right)}^{2n}} & 1 & 0\\ \frac{-j{\left(\tau_{1}\omega_{1}\right)}^{n}\;\sin\left(\frac{n\pi }{2}\right)}{1\;+\;2{\left(\tau_{1}\omega_{1}\right)}^{n}\;\cos\left(\frac{n\pi }{2}\right)\;+\;{\left(\tau_{1}\omega_{1}\right)}^{2n}} & \cdots & \frac{-j{\left(\tau_{N_{RQ}}\omega_{1}\right)}^{n}\;\sin\left(\frac{n\pi }{2}\right)}{1\;+\;2{\left(\tau_{N_{RQ}}\omega_{1}\right)}^{n}\;\cos\left(\frac{n\pi }{2}\right)\;+\;{\left(\tau_{N_{RQ}}\omega_{1}\right)}^{2n}} & 0 & \omega_{1}\\ \vdots & \ddots & \vdots & \vdots & \vdots \\ \frac{-j{\left(\tau_{1}\omega_{N_{EIS}}\right)}^{n}\;\sin\left(\frac{n\pi }{2}\right)}{1\;+\;2{\left(\tau_{1}\omega_{N_{EIS}}\right)}^{n}\;\cos\left(\frac{n\pi }{2}\right)\;+\;{\left(\tau_{1}\omega_{N_{EIS}}\right)}^{2n}} & \cdots & \frac{-j{\left(\tau_{N_{RQ}}\omega_{N_{EIS}}\right)}^{n}\;\sin\left(\frac{n\pi }{2}\right)}{1\;+\;2{\left(\tau_{N_{RQ}}\omega_{N_{EIS}}\right)}^{n}\;\cos\left(\frac{n\pi }{2}\right)\;+\;{\left(\tau_{N_{RQ}}\omega_{N_{EIS}}\right)}^{2n}} & 0 & \omega_{N_{EIS}} \end{array}\right]$$

Similarly, by employing a nonlinear least squares optimizer to minimize the objective function, the relaxation time distribution function based on the RQ element can be obtained, as shown in Figure S3. From the figure, it can be observed that the DRT obtained using the RQ-based kernel consists of several distinct Dirac-like pulses, resulting in a concise and interpretable structure. Compared with the traditional RC-based approach, which is highly sensitive to the selection of kernel function and regularization coefficient, the RQ-based DRT exhibits only slight variations in pulse amplitude and frequency position when these parameters change. This characteristic significantly improves the robustness and consistency of the relaxation time distribution. The enhanced stability reduces the likelihood of artificial peaks and enables more reliable extraction of electrochemical features, thereby facilitating a deeper understanding of the internal kinetic processes within the battery system.

#### Feature selection

To scientifically select features, the correlation coefficient method is employed to assess the relationship between features and labels, which is a filtering approach. The Minimum Redundancy Maximum Relevance (MRMR) method is used to identify the subset of features that are most strongly correlated with the final output, while ensuring minimal redundancy among the features. This approach takes into account both the correlation between features and labels, as well as the inter-correlation among the features themselves.

The pearson correlation coefficient is a commonly used statistical measure to assess the strength and direction of the linear relationship between two variables. It is widely applied in data analysis, machine learning, and other fields. The pearson correlation coefficient is defined as the ratio of the covariance of two variables (X, Y) to the product of their standard deviations, as shown in Equation 12:

(Equation 12) $$r=\frac{\sum \left(X_{i}-\bar{X}\right)\left(Y_{i}-\bar{Y}\right)}{\sqrt{\sum {\left(X_{i}-\bar{X}\right)}^{2}\sum {\left(Y_{i}-\bar{Y}\right)}^{2}}}$$

The pearson correlation coefficient ranges from −1 to 1. When the pearson correlation coefficient is −1, X and Y are perfectly negatively correlated; when it is 1, X and Y are perfectly positively correlated. The pearson correlation coefficient is a dimensionless measure, meaning it is not influenced by the units or scale of the variables, making it suitable for comparing variables with different units. However, the pearson correlation coefficient only measures linear relationships and is not applicable to nonlinear relationships. If two variables have a nonlinear relationship, the pearson correlation coefficient may underestimate the strength of this relationship. Therefore, even if the pearson correlation coefficient is 0, it cannot be concluded that the two variables are independent.

Through correlation coefficients, features that are strongly linearly related to the labels can be selected. However, redundant information between features may affect the regression performance to some extent. The MRMR algorithm addresses the mutual relationships between features and labels, as well as the redundancy between two features.^61^ The MRMR algorithm first considers maximum relevance, selecting a feature subset with n features that exhibit the highest correlation with the label. The maximum relevance searches for features that satisfy the following equation:

(Equation 13) $$\max\;D\left(X,y\right),D=\frac{1}{\left|X\right|}\sum_{x_{i}\in X}I\left(x_{i},y\right)$$

where, X is the set of features, y is labels, |X| is the number of features, $I\left(x_{i},y\right)$ is mutual information between features and labels, D is the average mutual information between all features and labels.

The mutual information between the feature and label can be calculated using the following equation:

(Equation 14) $$I\left(x_{i},y\right)=\int \int p\left(x_{i},y\right)\log\frac{p\left(x_{i},y\right)}{p\left(x_{i}\right)p\left(y\right)}dx_{i}dy$$

where, p() is probability density function.

The features selected through maximum relevance may exhibit redundancy, as there is a high degree of dependency between them. When two features are redundant, removing one does not significantly impact the prediction results. Therefore, the MRMR method also reduces redundancy by calculating the minimum redundancy, thus eliminating redundant features as follows:

(Equation 15) $$\min\;R\left(X\right),R=\frac{1}{{\left|X\right|}^{2}}\sum_{x_{i},x_{j}\in X}I\left(x_{i},x_{j}\right)$$

The maximum relevance D and minimum redundancy R are combined in the MRMR method. Here, an operator Φ(D,R) is defined to combine D and R, with the simplest combination being as follows:

(Equation 16) maxΦ(D,R),Φ=D−R

In practical applications, based on the defined operator Φ(D,R), an incremental search method can be used to obtain a near-optimal solution. Suppose the $S_{m-1}$ feature subset has already been obtained, and the m-th feature needs to be selected from the remaining feature subset $X-S_{m-1}$. Feature selection is performed by maximizing Φ(D,R), that is, by maximizing:

(Equation 17) $$\max_{x_{j}\in X-S_{m-1}}\left[I\left(x_{j},y\right)-\frac{1}{m-1}\sum_{x_{i}\in S_{m-1}}I\left(x_{i},x_{j}\right)\right]$$

The MRMR algorithm can quantify the importance of each feature, thereby ranking all the elements.

#### Regression algorithms for SOH estimation

Linear regression is the simplest and most popular regression tool. It assumes a linear relationship between the features X and the target y, meaning the target can be expressed as a weighted sum of the features, as shown in the following equation:

(Equation 18) $$y=w_{1}x_{1}+\cdots +w_{d}x_{d}+b$$

where, w is weight coefficients, b is bias, d is the number of features.

The goal of linear regression is to find a set of weight vector w and bias b such that the prediction error for new samples is minimized. To find this set of weights, we typically need to identify a measure of how well the model fits the data and use methods to update the model in order to improve prediction quality.

The loss function quantifies the difference between the actual value and the predicted value of the target. Typically, non-negative values are chosen for the loss, with smaller values indicating smaller loss, and a loss of 0 representing a perfect prediction. The most commonly used loss function in regression problems is the squared error function. When the predicted value for sample i is ${\hat{y}}^{\left(i\right)}$ and the corresponding true label is $y^{\left(i\right)}$, the squared error can be defined by the following formula:

(Equation 19) $$l^{i}\left(w,b\right)=\frac{1}{2}{\left({\hat{y}}^{\left(i\right)}-y^{\left(i\right)}\right)}^{2}$$

In order to measure the quality of the model across the entire dataset, we need to compute the average loss over the n samples in the training set:

(Equation 20) $$L\left(w,b\right)=\frac{1}{n}\sum_{i=1}^{n}l^{\left(i\right)}\left(w,b\right)$$

During model training, we aim to find a set of parameters $\left(w^{*},b^{*}\right)$ that minimize the total loss over all training samples, as shown in the following equation:

(Equation 21) $$w^{*},b^{*}=\arg\min L\left(w,b\right)$$

Gradient descent can be used in almost all deep learning models to improve prediction quality. It reduces error by continuously updating parameters in the direction of the loss function’s gradient. The simplest implementation method is to compute the derivative of the loss function with respect to the model parameters.

The limitation of linear models lies in their assumption of linearity in the transformation, meaning that when the weight is positive, an increase in any feature leads to an increase in the model’s output, and when the weight is negative, it causes a decrease in the model’s output. This assumption does not hold in some cases and can lead to significant prediction errors.

The MLP overcomes the limitations of linear models by adding one or more hidden layers to the network, enabling it to handle more general types of functional relationships.^62^ A typical MLP consists of an input layer, hidden layers, and an output layer. The input layer is at the forefront of the network and is responsible for handling complex input features. The hidden layers are positioned in the middle, and the output layer is at the end to produce the results. The input layer does not involve any computation, and the network’s output is achieved through the computations of the hidden and output layers.

Assume the dataset consists of n samples, each with d output features, denoted as $X\in R^{n\times d}$. For a single hidden-layer MLP with h hidden units, let $H\in R^{n\times h}$ represent the hidden layer variables. Since the hidden and output layers are fully connected, the hidden layer weights $W^{\left(1\right)}\in R^{d\times h}$, hidden layer bias $b^{\left(1\right)}\in R^{1\times h}$, output layer weights $W^{\left(2\right)}\in R^{h\times q}$, and output layer bias $b^{\left(2\right)}\in R^{1\times q}$ are defined. Therefore, the output of the hidden layer $O\in R^{n\times q}$ can be expressed as the following equation:

(Equation 22) $$\begin{array}{l} H=XW^{\left(1\right)}+b^{\left(1\right)}\\ O=HW^{\left(2\right)}+b^{\left(2\right)} \end{array}$$

To leverage the advantages of MLP, activation functions are used to apply nonlinear transformations to each hidden unit. Commonly used activation functions include the ReLU function, sigmoid function, and tanh function. MLP can capture complex interactions between inputs by using hidden neurons, which depend on the value of each input.

#### Model setup and training

In this study, we aim to achieve high-accuracy estimation of the SOH based on HIs extracted using the DRT algorithm with RQ elements. Given the practical challenges of acquiring EIS under fixed SOC conditions, we combine HIs obtained from different SOC levels into a unified training set. This strategy enhances the model’s adaptability across a wide SOC range. A 4-fold cross-validation approach is employed for model training. In each fold, the HIs from one SOC level are selected as the test set, while those from the remaining three SOC levels serve as the training set. The dataset division of 4-fold cross-validation is shown in Table S1. This ensures that data from each SOC level is used once for testing, thereby improving the credibility and generalizability of the model. Similarly, for the open-access dataset, we adopt the same 4-fold cross-validation strategy to evaluate the model’s adaptability to different battery samples.

The model used in this work is a simple MLP, which helps prevent overfitting and accelerates training. Due to the strong correlation between the DRT-extracted HIs and SOH, this lightweight model is sufficient to achieve high estimation accuracy. The rectified linear unit (ReLU) is used as the activation function for nonlinear transformation, and the Adam optimizer is employed to update network parameters. The learning rate is set to 0.01, and the model is trained for 1000 epochs.

### Quantification and statistical analysis

All the statistical analysis methods employed were implemented in Python. To evaluate the stability and robustness of the proposed models under different conditions, Figure 6 presents the mean relative error (MRE), root-mean-square error (RMSE), and the 90% confidence interval (CI) lengths calculated over ten repeated training runs. These metrics provide a comprehensive assessment of both prediction accuracy and the consistency of the model across multiple runs. Table 3 further summarizes the detailed values of RMSE, MRE, and 90% CI lengths under different SOC levels and across different battery cells, allowing for a direct comparison of model performance across various testing scenarios. The calculation formulas for these metrics are shown in Equation 23:

(Equation 23) $$\begin{array}{c} MRE=\frac{1}{\sum_{k=1}^{4}N_{k}}\sum_{k=1}^{4}\sum_{i=1}^{N_{k}}\left|\frac{{\hat{y}}_{i}-y_{i}}{y_{i}}\right|\\ RMSE=\sqrt{\frac{1}{\sum_{k=1}^{4}N_{k}}\sum_{k=1}^{4}\sum_{i=1}^{N_{k}}{\left({\hat{y}}_{i}-y_{i}\right)}^{2}}\\ L_{C1}=2\cdot t_{a/2,r-1}\cdot \frac{s}{\sqrt{r}} \end{array}$$

where, k denotes the kth fold in the cross-validation; N_k_ is the number of test samples in the kth folder; $y_{i}$ and ${\hat{y}}_{i}$ are the ground truth and predicted values of the ith test sample, respectively; r is number of repeated runs (in this case, r = 10); s is the standard deviation of the prediction values at each test point; $t_{a/2,r-1}$ the two-tailed critical value from the student’s t-distribution with r−1 degrees of freedom, corresponding to a confidence level of 90% (i.e., α = 0.1).

## References

[1] Li Z., Khajepour A., Song J. (2019). A comprehensive review of the key technologies for pure electric vehicles. Energy.

[2] Liu W., Placke T., Chau K.T. (2022). Overview of batteries and battery management for electric vehicles. Energy Rep..

[3] Harper G., Sommerville R., Kendrick E., Driscoll L., Slater P., Stolkin R., Walton A., Christensen P., Heidrich O., Lambert S. (2019). Recycling lithium-ion batteries from electric vehicles. Nature.

[4] Hasan M.K., Mahmud M., Ahasan Habib A.K.M., Motakabber S.M.A., Islam S. (2021). Review of electric vehicle energy storage and management system: Standards, issues, and challenges. J. Energy Storage.

[5] Zhang X., Li Z., Luo L., Fan Y., Du Z. (2022). A review on thermal management of lithium-ion batteries for electric vehicles. Energy.

[6] Zhang M., Liu Y., Li D., Cui X., Wang L., Li L., Wang K. (2023). Electrochemical Impedance Spectroscopy: A New Chapter in the Fast and Accurate Estimation of the State of Health for Lithium-Ion Batteries. Energies.

[7] Zhang C., Zhao S., Yang Z., He Y. (2023). A multi-fault diagnosis method for lithium-ion battery pack using curvilinear Manhattan distance evaluation and voltage difference analysis. J. Energy Storage.

[8] Tian H., Qin P., Li K., Zhao Z. (2020). A review of the state of health for lithium-ion batteries: Research status and suggestions. J. Clean. Prod..

[9] Liu Y., Wang L., Li D., Wang K. (2023). State-of-health estimation of lithium-ion batteries based on electrochemical impedance spectroscopy: a review. Prot. Control Mod. Power Syst..

[10] Ge M.F., Liu Y., Jiang X., Liu J. (2021). A review on state of health estimations and remaining useful life prognostics of lithium-ion batteries. Measurement.

[11] Luo K., Chen X., Zheng H., Shi Z. (2022). A review of deep learning approach to predicting the state of health and state of charge of lithium-ion batteries. J. Energy Chem..

[12] Lin Y.H., Ruan S.J., Chen Y.X., Li Y.F. (2023). Physics-informed deep learning for lithium-ion battery diagnostics using electrochemical impedance spectroscopy. Renew. Sustain. Energy Rev..

[13] Xiong R., Wang J., Shen W., Tian J., Mu H. (2021). Co-Estimation of State of Charge and Capacity for Lithium-Ion Batteries with Multi-Stage Model Fusion Method. Engineering.

[14] Chen S., Zhang Q., Wang F., Wang D., He Z. (2024). An electrochemical-thermal-aging effects coupled model for lithium-ion batteries performance simulation and state of health estimation. Appl. Therm. Eng..

[15] Xiong R., Sun Y., Wang C., Tian J., Chen X., Li H., Zhang Q. (2023). A data-driven method for extracting aging features to accurately predict the battery health. Energy Storage Mater..

[16] Wang P., Peng X., Ze C. (2022). State-of-health estimation for lithium-ion batteries using differential thermal voltammetry and Gaussian process regression. J. Power Electron..

[17] Tian Y., Wen J., Yang Y., Shi Y., Zeng J. (2022). State-of-Health Prediction of Lithium-Ion Batteries Based on CNN-BiLSTM-AM. Batteries.

[18] Fang D., Wu W., Li J., Yuan W., Liu T., Dai C., Wang Z., Zhao M. (2023). Performance simulation method and state of health estimation for lithium-ion batteries based on aging-effect coupling model. Green Energy and Intelligent Transportation.

[19] Pastor-Fernández C., Uddin K., Chouchelamane G.H., Widanage W.D., Marco J. (2017). A Comparison between Electrochemical Impedance Spectroscopy and Incremental Capacity-Differential Voltage as Li-ion Diagnostic Techniques to Identify and Quantify the Effects of Degradation Modes within Battery Management Systems. J. Power Sources.

[20] Deng Z., Lin X., Cai J., Hu X. (2022). Battery health estimation with degradation pattern recognition and transfer learning. J. Power Sources.

[21] Li W., Fan Y., Ringbeck F., Jöst D., Sauer D.U. (2022). Unlocking electrochemical model-based online power prediction for lithium-ion batteries via Gaussian process regression. Appl. Energy.

[22] Chen L., Xie S., Lopes A.M., Li H., Bao X., Zhang C., Li P. (2024). A new SOH estimation method for Lithium-ion batteries based on model-data-fusion. Energy.

[23] Wang J., Zhao R., Huang Q.A., Wang J., Fu Y., Li W., Bai Y., Zhao Y., Li X., Zhang J. (2023). High-efficient prediction of state of health for lithium-ion battery based on AC impedance feature tuned with Gaussian process regression. J. Power Sources.

[24] Chen X., Liu X., Shen X., Zhang Q. (2021). Applying Machine Learning to Rechargeable Batteries: From the Microscale to the Macroscale. Angew. Chem. Int. Ed. Engl..

[25] Khaire U.M., Dhanalakshmi R. (2022). Stability of feature selection algorithm: A review. J. King Saud Univ. Comput. Inf. Sci..

[26] Shen S., Sadoughi M., Li M., Wang Z., Hu C. (2020). Deep convolutional neural networks with ensemble learning and transfer learning for capacity estimation of lithium-ion batteries. Appl. Energy.

[27] Qian C., Xu B., Chang L., Sun B., Feng Q., Yang D., Ren Y., Wang Z. (2021). Convolutional neural network based capacity estimation using random segments of the charging curves for lithium-ion batteries. Energy.

[28] Zhang C., Tu L., Yang Z., Du B., Zhou Z., Wu J., Chen L. (2025). A CMMOG-based lithium-battery SOH estimation method using multi-task learning framework. J. Energy Storage.

[29] Samad N.A., Kim Y., Siegel J.B., Stefanopoulou A.G. (2016). Battery Capacity Fading Estimation Using a Force-Based Incremental Capacity Analysis. J. Electrochem. Soc..

[30] Jiang B., Zhu J., Wang X., Wei X., Shang W., Dai H. (2022). A comparative study of different features extracted from electrochemical impedance spectroscopy in state of health estimation for lithium-ion batteries. Appl. Energy.

[31] Knehr K.W., Hodson T., Bommier C., Davies G., Kim A., Steingart D.A. (2018). Understanding Full-Cell Evolution and Non-chemical Electrode Crosstalk of Li-Ion Batteries. Joule.

[32] Zhu J., Wang Y., Huang Y., Bhushan Gopaluni R., Cao Y., Heere M., Mühlbauer M.J., Mereacre L., Dai H., Liu X. (2022). Data-driven capacity estimation of commercial lithium-ion batteries from voltage relaxation. Nat. Commun..

[33] Li X., Yuan C., Li X., Wang Z. (2020). State of health estimation for Li-Ion battery using incremental capacity analysis and Gaussian process regression. Energy.

[34] Tian J., Xiong R., Shen W. (2020). State-of-Health Estimation Based on Differential Temperature for Lithium Ion Batteries. IEEE Trans. Power Electron..

[35] Bao X., Chen L., Lopes A.M., Li X., Xie S., Li P., Chen Y. (2023). Hybrid deep neural network with dimension attention for state-of-health estimation of Lithium-ion Batteries. Energy.

[36] Zhang C., Luo L., Yang Z., Du B., Zhou Z., Wu J., Chen L. (2024). Flexible method for estimating the state of health of lithium-ion batteries using partial charging segments. Energy.

[37] Liu J., Duan Q., Ma M., Zhao C., Sun J., Wang Q. (2020). Aging mechanisms and thermal stability of aged commercial 18650 lithium ion battery induced by slight overcharging cycling. J. Power Sources.

[38] Stroe D.I., Schaltz E. (2020). Lithium-Ion Battery State-of-Health Estimation Using the Incremental Capacity Analysis Technique. IEEE Trans. Ind. Appl..

[39] Schaltz E., Stroe D.I., Norregaard K., Ingvardsen L.S., Christensen A. (2021). Incremental Capacity Analysis Applied on Electric Vehicles for Battery State-of-Health Estimation. IEEE Trans. Ind. Appl..

[40] Lu Y., Zhao C.Z., Huang J.Q., Zhang Q. (2022). The timescale identification decoupling complicated kinetic processes in lithium batteries. Joule.

[41] Zhu Y., Jiang B., Zhu J., Wang X., Wang R., Wei X., Dai H. (2023). Adaptive state of health estimation for lithium-ion batteries using impedance-based timescale information and ensemble learning. Energy.

[42] Zhang Q., Wang D., Schaltz E., Stroe D.I., Gismero A., Yang B. (2022). Degradation mechanism analysis and State-of-Health estimation for lithium-ion batteries based on distribution of relaxation times. J. Energy Storage.

[43] Wang X., Wei X., Chen Q., Dai H. (2021). A Novel System for Measuring Alternating Current Impedance Spectra of Series-Connected Lithium-Ion Batteries with a High-Power Dual Active Bridge Converter and Distributed Sampling Units. IEEE Trans. Ind. Electron..

[44] Yang B., Wang D., Yu B., Wang F., Chen S., Sun X., Dong H. (2024). Research on online passive electrochemical impedance spectroscopy and its outlook in battery management. Appl. Energy.

[45] Fu Y., Xu J., Shi M., Mei X. (2022). A Fast Impedance Calculation-Based Battery State-of-Health Estimation Method. IEEE Trans. Ind. Electron..

[46] Babaeiyazdi I., Rezaei-Zare A., Shokrzadeh S. (2021). State of charge prediction of EV Li-ion batteries using EIS: A machine learning approach. Energy.

[47] Galeotti M., Cinà L., Giammanco C., Cordiner S., Di Carlo A. (2015). Performance analysis and SOH (state of health) evaluation of lithium polymer batteries through electrochemical impedance spectroscopy. Energy.

[48] Gavrilyuk A.L., Osinkin D.A., Bronin D.I. (2017). The use of Tikhonov regularization method for calculating the distribution function of relaxation times in impedance spectroscopy. Russ. J. Electrochem..

[49] Dierickx S., Weber A., Ivers-Tiffée E. (2020). How the distribution of relaxation times enhances complex equivalent circuit models for fuel cells. Electrochim. Acta.

[50] Li X., Ahmadi M., Collins L., Kalinin S.V. (2019). Deconvolving distribution of relaxation times, resistances and inductance from electrochemical impedance spectroscopy via statistical model selection: Exploiting structural-sparsity regularization and data-driven parameter tuning. Electrochim. Acta.

[51] Zhang Y., Chen Y., Li M., Yan M., Ni M., Xia C. (2016). A high-precision approach to reconstruct distribution of relaxation times from electrochemical impedance spectroscopy. J. Power Sources.

[52] Gavrilyuk A.L., Osinkin D.A., Bronin D.I. (2020). On a variation of the Tikhonov regularization method for calculating the distribution function of relaxation times in impedance spectroscopy. Electrochim. Acta.

[53] Zhang Y., Tang Q., Zhang Y., Wang J., Stimming U., Lee A.A. (2020). Identifying degradation patterns of lithium ion batteries from impedance spectroscopy using machine learning. Nat. Commun..

[54] Xia B., Qin Z., Fu H. (2024). Rapid estimation of battery state of health using partial electrochemical impedance spectra and interpretable machine learning. J. Power Sources.

[55] Obregon J., Han Y.R., Ho C.W., Mouraliraman D., Lee C.W., Jung J.Y. (2023). Convolutional autoencoder-based SOH estimation of lithium-ion batteries using electrochemical impedance spectroscopy. J. Energy Storage.

[56] Liu Z., Sun Y., Li Y., Liu Y., Chen Y., Zhang Y. (2024). Lithium-ion battery health prognosis via electrochemical impedance spectroscopy using CNN-BiLSTM model. J. Mater. Inf..

[57] Wang X., Wei X., Dai H. (2019). Estimation of state of health of lithium-ion batteries based on charge transfer resistance considering different temperature and state of charge. J. Energy Storage.

[58] Zhang K., Xiong R., Qu S., Zhang B., Shen W. (2024). Electrochemical Impedance Spectroscopy: A Novel High-Power Measurement Technique for Onboard Batteries Using Full-Bridge Conversion. IEEE Trans. Transp. Electrific..

[59] Wang L., Zeng X., Chen L., Lv L., Liao L., Jiang J. (2025). An Active Equalization Method for Cascade Utilization Lithium-Ion Battery Pack With Online Measurement of Electrochemical Impedance Spectroscopy. J. Electrochem. En. Conv. Stor..

[60] Schmidt J.P., Chrobak T., Ender M., Illig J., Klotz D., Ivers-Tiffée E. (2011). Studies on LiFePO4 as cathode material using impedance spectroscopy. J. Power Sources.

[61] Liang Y., Niu D., Hong W.C. (2019). Short term load forecasting based on feature extraction and improved general regression neural network model. Energy.

[62] Lee S., Han S., Han K.H., Kim Y., Agarwal S., Hariharan K.S., Oh B., Yoon J. (2021). Diagnosing various failures of lithium-ion batteries using artificial neural network enhanced by likelihood mapping. J. Energy Storage.

