# Supplementary material for: SOH estimation for lithium-ion batteries using the distribution of relaxation time and feature optimized multilayer perceptron
Source: iScience. 2025 Aug 26;28(9):113443. doi: 10.1016/j.isci.2025.113443 (PMC12496215; doi:10.1016/j.isci.2025.113443)
Supplement: Document S1. Figures S1–S3 and Table S1 [file mmc1.pdf]

iScience, Volume *28*

Supplemental information

SOH estimation for lithium-ion batteries using the distribution of relaxation time and feature optimized multilayer perceptron

Fang Wang, Shiqiang Liu, Shiqin Chen, Qi Zhang, Dafang Wang, Xiaole Ma, and Xiaoqian Dai

Supplemental information

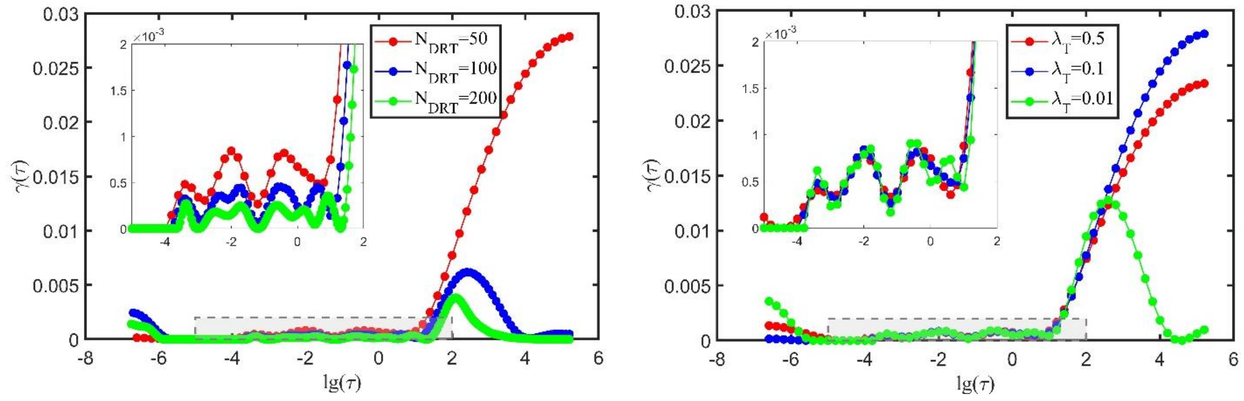

(a) N_{DRT} (b) λ_T

Fig. S1. The influence of different selections of N_{DRT} and λ_T on the relaxation time distribution function

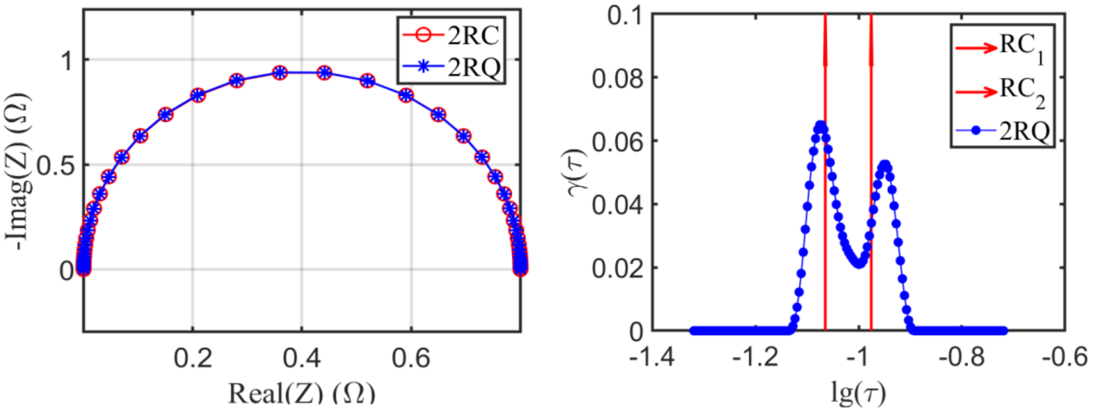

(a)The impedance spectrum (b)The relaxation times distribution

Fig. S2. The impedance spectrum and relaxation times distribution of RC element and RQ element

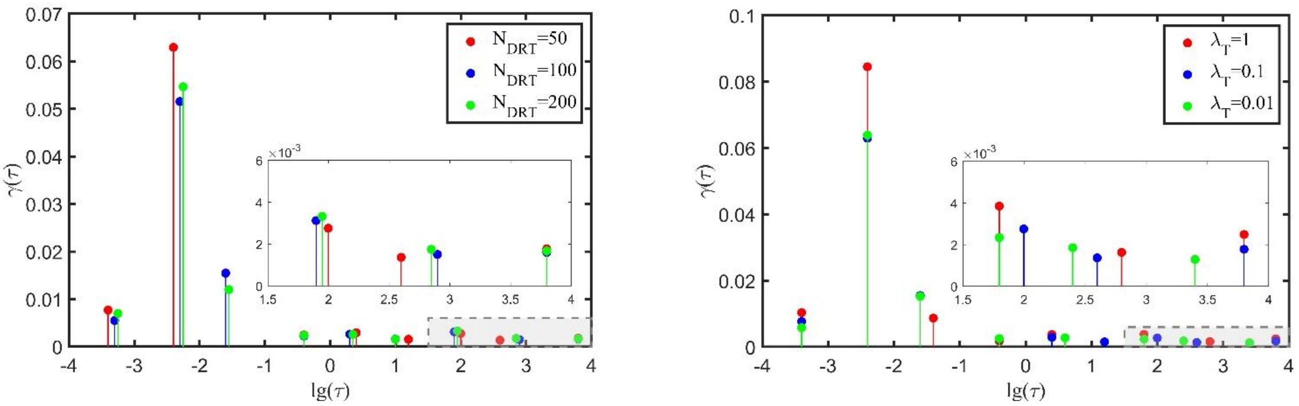

(a) N_{DRT} (b) λ_T

Fig. S3. DRT results based on the RQ element

Table S1

Parameters of the batteries under test

Samples	Dataset	Fold1	Fold2	Fold3	Fold4
S1, S2	Training set	35%SOC, 55%SOC, 75%SOC	15%SOC, 55%SOC, 75%SOC	15%SOC, 35%SOC, 75%SOC	15%SOC, 35%SOC, 55%SOC
	Testing set	15%SOC	35%SOC	55%SOC	75%SOC
S3	Training set	S3_2, S3_3, S3_4	S3_1, S3_3, S3_4	S3_1, S3_2, S3_4	S3_1, S3_2, S3_3
	Testing set	S3_1	S3_2	S3_3	S3_4
